# Effects of Argon Gas Plasma Treatment on Biocompatibility of Nanostructured Titanium

**DOI:** 10.3390/ijms25010149

**Published:** 2023-12-21

**Authors:** Rina Hayashi, Seiji Takao, Satoshi Komasa, Tohru Sekino, Tetsuji Kusumoto, Kenji Maekawa

**Affiliations:** 1 Department of Removable Prosthodontics and Occlusion, Osaka Dental University, 8-1 Kuzuha-hanazono-cho, Hirakata 573-1121, Osaka, Japan; hayashi-r@cc.osaka-dent.ac.jp (R.H.); takao-s@cc.osaka-dent.ac.jp (S.T.); maekawa-k@cc.osaka-dent.ac.jp (K.M.); 2 Department of Advanced Hard Materials, SANKEN (The Institute of Scientific and Industrial Research), Osaka University, 8-1 Mihogaoka, Ibaraki 567-0047, Osaka, Japan; sekino@sanken.osaka-u.ac.jp; 3 Department of Oral Health Engineering, Faculty of Health Sciences, Osaka Dental University, 1-4-4, Makino-honmachi, Hirakata 573-1121, Osaka, Japan; kusumoto@cc.osaka-dent.ac.jp

**Keywords:** argon plasma treatment, titania nanosheets, reactive oxygen species, hard tissue differentiation, rat bone marrow, bone formation, simulated body fluid

## Abstract

In this study, we applied argon plasma treatment to titanium surfaces with nanostructures deposited by concentrated alkali treatment and investigated the effects on the surface of the material and the tissue surrounding an implant site. The results showed that the treatment with argon plasma removed carbon contaminants and increased the surface energy of the material while the nanoscale network structure deposited on the titanium surface remained in place. Reactive oxygen species reduced the oxidative stress of bone marrow cells on the treated titanium surface, creating a favorable environment for cell proliferation. Good results were observed in vitro evaluations using rat bone marrow cells. The group treated with argon plasma exhibited the highest apatite formation in experiments using simulated body fluids. The results of in vivo evaluation using rat femurs revealed that the treatment improved the amount of new bone formation around an implant. Thus, the results demonstrate that argon plasma treatment enhances the ability of nanostructured titanium surfaces to induce hard tissue differentiation and supports new bone formation around an implant site.

## 1. Introduction

Improving the performance of dental implants by facilitating and maintaining their attachment to hard and soft tissues has attracted considerable attention in terms of expanding their range of applications [1,2]. Titanium and its alloys have been widely utilized as implant materials because of their excellent biocompatibility [3,4]. Additionally, the thin oxide layer deposited on the titanium surface has been shown to exhibit excellent corrosion resistance [1]. The long-term clinical success rate of such implants has been reported as 89.23% at the 10-year follow-up period [5]. Early osseointegration, long-term stability of the bone-implant interface, and reduction of peri-implantitis are necessary to increase the long-term success rate of such implants, and the mechanical and chemical properties of the material surface play a vital role in implant osseointegration [6,7,8,9].

The surface properties of implant materials have a remarkable impact on the speed and coverage of their ability to induce hard tissue differentiation [10,11]. Vandrovcova et al. [12] reported that modifying the surface of a titanium implant improved the initial adhesion ability of rat bone marrow (RBM) cells, enhanced their ability to induce hard tissue differentiation, and ensured secondary stability. Various methods have been developed to modify the titanium surfaces, which have primarily focused on the surface structure and the properties of biomaterials for enhancing the osseointegration of dental implants [1,2,3]. The results of our previous studies have shown that the implant surface characteristics have a remarkable impact on cell adhesion and proliferation, as well as on cell differentiation and osteogenesis-related gene expression [13,14,15]. Titania nanosheets (TNSs), i.e., nanometer-scale structures, can be formed on the material surface by treating titanium with alkali to create a rough surface at nanoscale. Furthermore, the precipitated nano-modified titanium surface is useful for improving the initial adhesion ability of rat bone marrow cells (RBMCs) and inducing hard tissue differentiation [16,17,18,19]. Further, TNSs have been reported to be more hydrophilic than untreated titanium surfaces [13,14]. Therefore, they can be considered valuable for the initial adhesion of proteins and RBMCs, as well as for hydroxyapatite formation. These factors result in excellent bone conduction ability. However, their clinical application has been limited by several issues such as the high rate of bacterial adhesion, risk of peri-implantitis, and insufficient amount of new bone formation in vivo [15]. Hence, generating a superhydrophilic material surface emerges as a potential solution to resolve these concerns.

The surface energy and wettability of titanium change owing to the deposition of carbon on the material surface, leading to a phenomenon called “aging” [20,21]. Carbon adheres to the titanium surface, reducing its hydrophilicity and the adsorption of proteins involved in osseointegration, which decreases the contact rate between the bone and the implant. The amount of carbon on the material surface is reduced by hydrophilic treatment of the surface, which increases the surface energy [22,23]. Physical and chemical treatment methods have been proposed as superhydrophilic treatments for titanium surfaces. Physical treatment methods such as atmospheric pressure plasma and ultraviolet (UV) treatment do not change the surface shape [24,25]. The superhydrophilic properties are imparted by decomposing the hydrocarbons on the surface and introducing hydroxyl groups [26,27,28]. No difference was observed between atmospheric-pressure plasma and UV treatments. It was proposed that the hydrocarbon adsorbed on the titanium surface decomposed, and hydroxyl groups were introduced onto the titanium oxide surface, resulting in superhydrophilicity [29,30,31]. Matsumoto et al. used a quartz crystal microbalance (QCM) device to compare the effects of atmospheric-pressure plasma and UV treatments on titanium surfaces [32] and inferred that both the treatments imparted hydrophilicity to the titanium surface, which increased the amount of protein and cell attachment, indicating that atmospheric pressure plasma treatment could be effective. This could be attributed to the higher energy of the plasma treatment compared to UV irradiation, and a superhydrophilic surface could be obtained in an extremely brief time. Consequently, we focused on atmospheric-pressure plasma treatment and investigated its effect on the bone conductivity of titanium and zirconia. Ujino et al. demonstrated that the in vitro and in vivo atmospheric-pressure plasma treatment of titanium surfaces was effective in improving the adhesion ability of RBMCs and inducing hard tissue differentiation [31]. Further, atmospheric-pressure plasma treatment of the zirconia material surface was found to be as effective as titanium in improving bone conduction performance, as reported in our previous studies [33,34]. Several studies have revealed that rendering the material surface superhydrophilic decreases the carbon and oxidative stress, thus affecting the behavior of RBMCs [29,35]. In this study, we used a small and lightweight plasma device. Therefore, this approach can be highly useful in clinical applications. Additionally, the device was equipped with a gas nozzle, which enabled it to contain various gases. Higher bone conductivity was imparted to the titanium surface by including argon gas in the atmospheric-pressure plasma as compared to that using only atmospheric-pressure plasma. Hence, implant materials with higher osteoconductivity can be developed by subjecting the nanostructured titanium surface to atmospheric-pressure plasma treatment containing argon gas. Therefore, we compared and examined the effects of atmospheric-pressure plasma treatment containing argon gas on titanium surfaces treated with an alkali in this study and considered its subsequent effects on bone conduction performance.

## 2. Results

### 2.1. Surface Characterization

**Figure 1:**
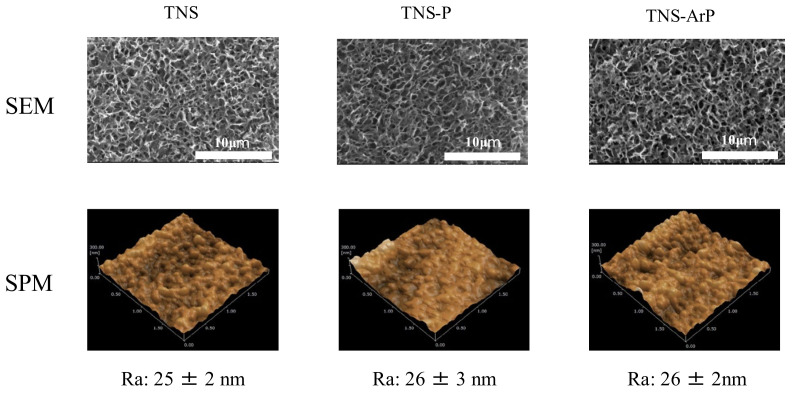
Scanning electron microscopy (SEM) and scanning probe microscopy (SPM) micrographs of TNS, TNS-P, and TNS-ArP groups.

**Figure 2:**
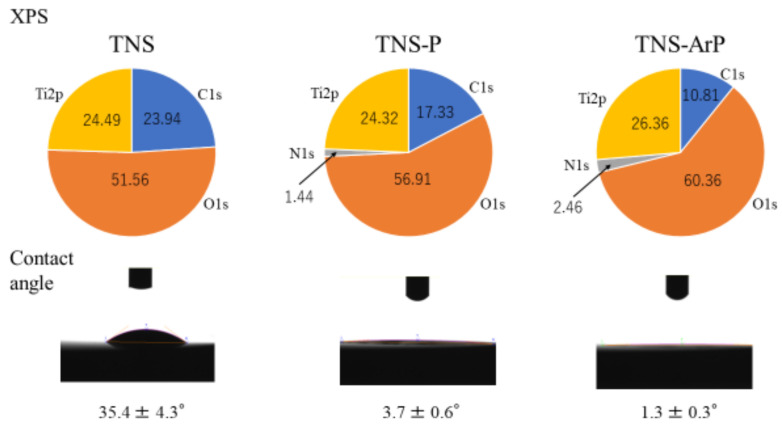
X-ray photoelectron spectroscopy (XPS) analysis and contact angle of TNS, TNS-P, and TNS-ArP groups. (Titanium; Ti2p, carbon; C1s, oxygen; O1s, nitrogen; N1s).

Figure 1 shows scanning electron microscopy (SEM) micrographs of titanium samples, which show a well-interconnected and homogeneous nanoporous network structure with an average diameter of 50–100 nm on the titanium surface after alkali treatment. No change was observed on the titanium surface owing to the TNS, TNS-P and TNS-ArP groups (Figure 1). Further, an analysis using scanning probe microscopy (SPM) showed no change in the surface roughness of test disks (Figure 1). Analysis by X-ray photoelectron spectroscopy (XPS) suggested an increase in the O1s peak intensity and a decrease in the C1s peak height on the titanium surface, which could be attributed to the plasma treatment (Figure 2). The C1s peak of the TNS-ArP group showed the lowest intensity. The contact angle of the material surface was reduced by the plasma treatment, which exhibited the lowest value in the TNS-ArP group (Figure 2). Further, the number of polar surface components substantially increased for both the TNS-P (74.2 ± 0.9 mJ/m^2^) and TNS-ArP (77.1 ± 1.2 mJ/m^2^) samples, which exhibited higher surface energies compared to a control group (60.2 ± 5.2 mJ/m^2^). The hydrogen bond components increased owing to plasma treatment and were highest in the TNS-ArP group.

### 2.2. Evaluation of Protein Adsorption on the Plasma and Nano-Modified Titanium Surface

**Figure 3:**
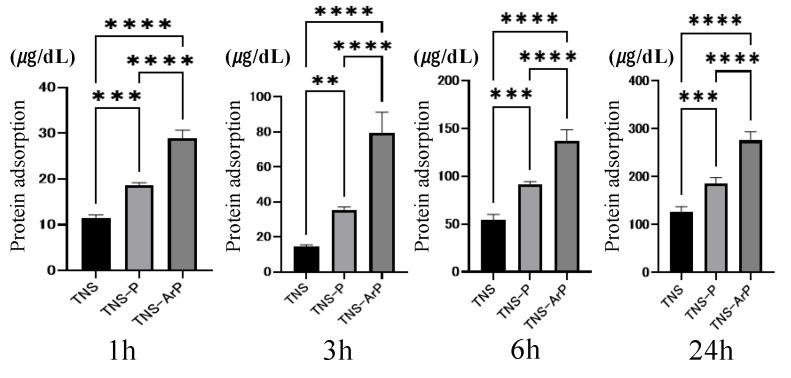
The adhesion examination results of BSA with the three groups. (*n* = 4, ** *p* < 0.01, *** *p* < 0.001, **** *p* < 0.0001). It was evaluated 1, 3, 6, and 24 h after drops of albumin were placed on the various samples.

Bovine serum albumin (BSA) adhesion was examined in three groups at 1, 3, 6, and 24 h after cell seeding (Figure 3). The BSA adsorption was considerably higher in the test group compared to the control group. Additionally, the highest value was observed for the TNS-ArP group.

### 2.3. Effects of Plasma and Nano-Modified Titanium Surfaces on Cell Adhesion and Morphology in RBMCs

**Figure 4:**
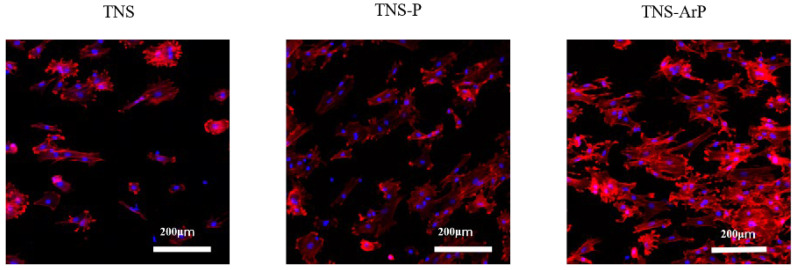
Morphologies of RBMCs on the titanium surfaces of all three groups after 24 h of culture observed using a confocal laser scanning microscope.

**Figure 5:**
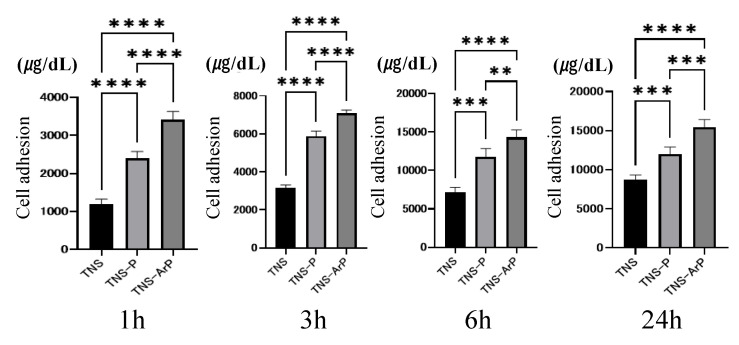
Number of adhered RBMCs for the three groups at various time intervals. (*n* = 4, ** *p* < 0.01, *** *p* < 0.001, **** *p* < 0.0001). It was evaluated 1, 3, 6, and 24 h after drops of RBMCs were placed on the various samples.

Fluorescence microscopy was used to determine the morphology of RBMCs on the titanium surface after 24 h of culture (Figure 4). The RBMCs adhered to the surfaces of the materials in all three groups. RBMCs adhesion was examined in three groups at 1, 3, 6, and 24 h after cell seeding (Figure 5). The number of adhered RBMCs after plasma treatment of the material surface was observed to be considerably higher than that in the control group, as shown. Additionally, the highest value of the number of adhered RBMCs among all three groups was observed for the TNS-ArP group.

### 2.4. In Vitro Argon Plasma Treatment-Induced Bone Differentiation on the Nano-Modified Titanium Surface

**Figure 6:**
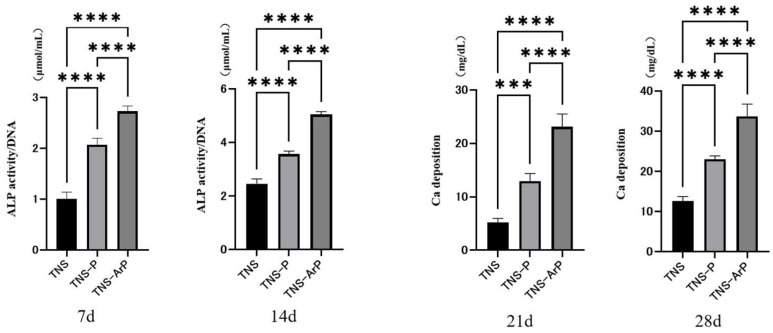
ALP activity in bone marrow cells at days 7 and 14 for all three groups. Calcium deposition on the material surface at days 21 and 28 after culture incubation for all three groups. (*n* = 4, *** *p* < 0.001, **** *p* < 0.0001).

**Figure 7:**
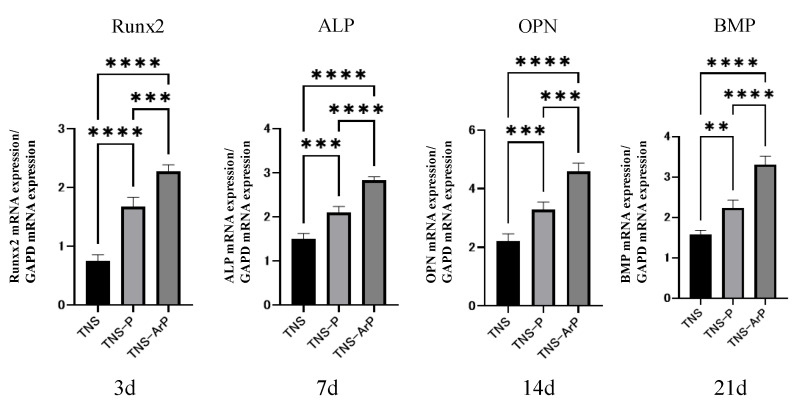
Gene expression related to the induction of hard tissue differentiation on the material surfaces of the samples in the test and control groups was analyzed. (*n* = 4, ** *p* < 0.01, *** *p* < 0.001, **** *p* < 0.0001). Gene expression levels related to the ability to induce hard tissue differentiation were analyzed 3, 7, 14, and 21 days after RBMCs were dropped on the surface of the material in each group. (Runx2 mRNA expression after 3 days, ALP mRNA expression after 7 days, OPN mRNA expression after 14 days, BMP mRNA expression after 21 days).

Bone differentiation on the nano-modified titanium surface was studied for all the groups. Among the three groups, alkaline phosphatase (ALP) activity in bone marrow cells 7 and 14 d after the start of culture was the highest on the material surface of the TNS-ArP group (Figure 6). Additionally, the amount of calcium deposited on the material surface 21 and 28 d after culture incubation was highest for the TNS-ArP group. Gene expression related to the induction of hard tissue differentiation on the material surfaces of the samples in the test and control groups was analyzed (Figure 7). The assay was performed at specific time intervals for each gene. A remarkably higher gene expression was achieved on the material surfaces of the samples in the TNS-ArP group as compared to that for the rest of the groups at all measured time intervals.

### 2.5. Cell Intracellular Reactive Oxygen Species (ROS) Level of RBMCs on Nano-Modified Titanium Surface

**Figure 8:**
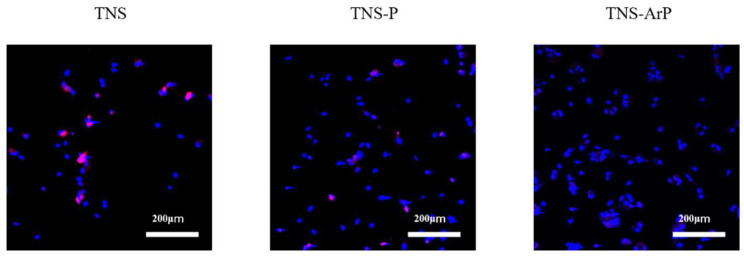
Fluorescence microscopy images of RBMCs on the nano-modified titanium surface for all the groups showing levels of intracellular ROS.

The RBMCs on the control group exhibited the highest levels of intracellular ROS among the three groups, whereas the lowest ROS levels were observed in the TNS-ArP group, indicating that argon plasma treatment enhanced the antioxidant properties of nano-modified titanium surfaces (Figure 8).

### 2.6. Simulated Body Fluid Immersion Experiment

**Figure 9:**
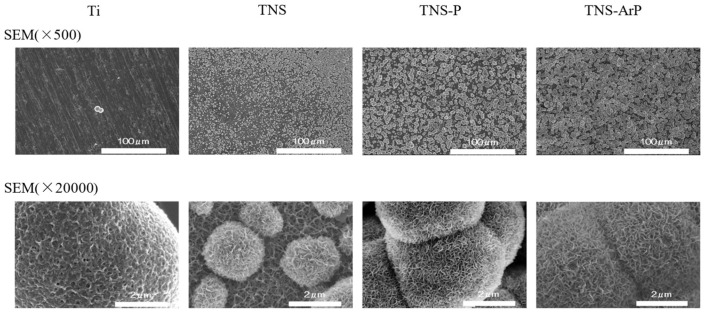
SEM results for Ti, TNS, TNS-P, and TNS-ArP groups at ×500 and ×20,000 magnification. (*n* = 8).

**Figure 10:**
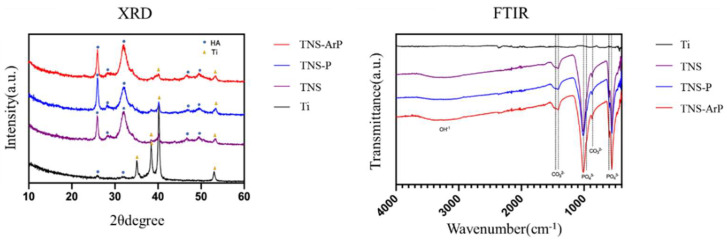
XRD patterns and FTIR spectra for Ti, TNS, TNS-P, and TNS-ArP groups. (*n* = 8). Dots indicate hydroxyapatite and triangles indicate titanium.

The results obtained using SEM revealed that a small amount of apatite was formed on the titanium surface (Figure 9). Furthermore, when comparing the TNS, TNS-P, and TNS-ArP groups at ×500 magnification, the amount of apatite formed was the lowest in the TNS group, moderate in the TNS-P group, and highest in the TNS-ArP group (Figure 9). Additionally, under high magnification (×20,000), the shape of the apatite was observed to differ on the titanium surfaces of the TNS, TNS-P, and TNS-ArP groups. Moreover, comparing the TNS, TNS-P, and TNS-ArP groups revealed that the apatite shape was smallest in the TNS and largest in the TNS-ArP group. X-ray diffraction (XRD) profiles showed a characteristic peak for apatite in the control and experimental groups, which was barely observed on the titanium surface (Figure 10).

Additionally, the measurement collected using Fourier transform infrared (FTIR) spectroscopy revealed the presence of apatite, hydroxide, and phosphate in the control and experimental groups, which were absent on the titanium surface (Figure 10).

### 2.7. Evaluation of the Amount of New Bone Formation in the Tissue Surrounding the Nano-Modified Titanium Implant Placement In Vivo

**Figure 11:**
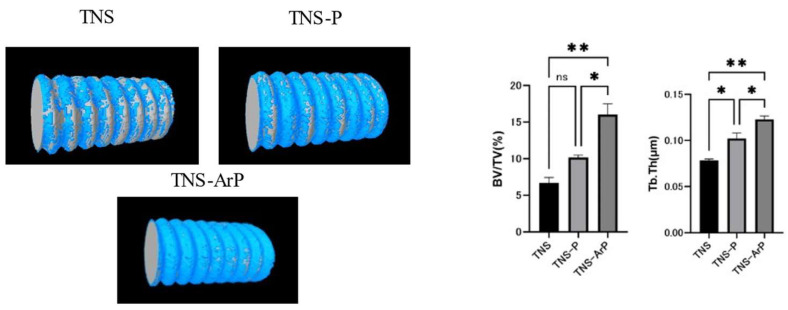
Trabecular microarchitecture, the ratio of bone mass to total mass (BV/TV), and the average trabecular thickness (Tb.Th) for the TNS, TNS-P, and TNS-ArP groups. (*n* = 8, * *p* < 0.05, ** *p* < 0.01, ns: not significant).

**Figure 12:**
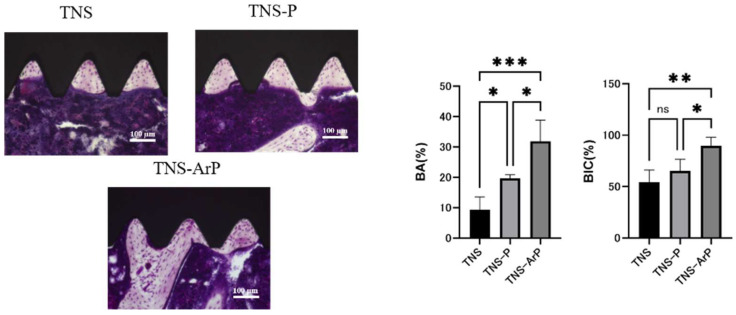
The amount of new bone formation was confirmed based on the longitudinal sections. Bone morphometric analysis showing that the bone area ratio (BA) and bone-to-implant contact (BIC) on the material surfaces of all three groups. (*n* = 8, * *p* < 0.05, ** *p* < 0.01, *** *p* < 0.001, ns: not significant).

The trabecular microarchitectures for all the groups, which were observed to be more prominent on the material surfaces of the test groups than on that of the control group (Figure 11). Furthermore, the ratio of bone mass to total mass (BV/TV) and the average trabecular thickness (Tb.Th) were the highest for the material surface of the TNS-ArP group among all three groups. These implants promoted osteogenic activity. The amount of new bone formation was determined using longitudinal sections (Figure 12). Among all three groups, a greater amount of newly formed bone was observed around the implants in the TNS-ArP group. Bone morphometric analysis showed that the bone area ratio (BA) and bone-to-implant contact (BIC) were the highest on the material surface of the TNS-ArP group among the three groups.

## 3. Discussion

We evaluated the effects of atmospheric-pressure plasma treatment on titanium surfaces. Upon irradiation with oxygen radicals via atmospheric-pressure plasma treatment, the carbon compounds combine with the radicals on the titanium surface, forming carbon dioxide, which was decomposed and removed. Therefore, our results confirm that titanium restores its original surface activity, induces the attachment and proliferation of proteins and RBMCs, and promotes bone formation. Argon gas, which is known to be inert, chemically unreactive to other elements, and exhibit a high sputtering rate, is sometimes used to generate plasma in a vacuum device. Argon gas is chemically unreactive, therefore, it can be used to suppress damage to the electrodes that come in contact with the plasma. We reported previously that treating titanium surfaces with concentrated alkali precipitates nanostructures and forms a network structure to which RBMCs can easily adhere, thus improving biocompatibility [13]. Therefore, it was hypothesized that atmospheric-pressure plasma treatment containing argon gas could improve the ability to induce hard tissue differentiation while maintaining the characteristics of the nanostructures deposited on the titanium surface. In the present work, we investigated the effects of atmospheric-pressure plasma treatment containing argon gas on the bone conductivity of nanomodified titanium surfaces. In vitro and in vivo evaluations were conducted using Sprague Dawley (SD) rats, and the apatite formation behavior was investigated in simulated body fluids.

SEM and SPM analyses revealed a nanoscale network structure on the titanium surface regardless of the plasma treatment. Previous reports have established that the surface roughness of this nanostructure is useful for improving the adhesion ability of RBMCs. It was observed that neither plasma treatment destroyed the nanostructures formed on the titanium surface, thereby maintaining the effectiveness of the argon gas-containing plasma, which is consistent with previous reports [36,37,38,39,40]. The production of many high-energy species through energy transfer reactions in a low-temperature plasma environment containing argon and oxygen in a specific ratio led to high surface energy on the nano-modified titanium, demonstrating that nano-modified titanium’s surface energy can be enhanced by atmospheric-pressure plasma treatment that contains argon gas. The wettability of the material surface was improved, leading to the lowest contact angle for the argon plasma treatment group and improved surface energy. Superhydrophilic surfaces have been observed in plasmas generated with argon as the input gas [41,42]. However, XPS analysis verified that the material surface’s chemical composition changed following the argon gas-containing plasma treatment. After plasma treatment, the amount of carbon decreased relative to the nano-modified titanium surface, and a significant number of polar oxygen groups formed; this phenomenon may be related to the increased hydrophilicity of the nano-modified titanium surface. The implant surface layer’s C–H bonds are broken by plasma treatment, which produces radicals and has enough activity to combat ROS on the material surface. The presence of oxygen and nitrogen on the material surface is reported to be associated with the induction of myeloid cell differentiation. Implant treatment always involves surgical procedures, leading to oxidative stress due to inflammation around the implant. However, excessive amounts cause delayed cell apoptosis, tissue healing, and implant failure. Therefore, suppression of ROS generation is essential to successful implant treatment. Carbon is found because carbon-containing atmospheric components inevitably adhere to these surfaces. However, the adsorption of carbon on the surface reduces biological activity; therefore, carbon must be reduced to improve activity [43,44,45]. The material’s surface acquires active oxygen due to treatment with argon plasma. In this study, argon plasma treatment of the nanostructured titanium surface suppressed the formation of oxidative stress on the material surface according to the ROS evaluation using RBMCs.

Immersion experiments in simulated body fluids have been reported as an effective methodology for evaluating the osteogenic ability of biomaterials [46,47,48,49]. Carbonate-containing apatite precipitates on the titanium surface upon immersion in Hank’s solution. In this study, the precipitation of hydroxyapatite crystals was observed on the nano-modified titanium surface. An apatite layer similar to that of bone is formed by immersing titanium with an alkali solution in a simulated body fluid with an inorganic ion concentration approximately equal to that of human body fluid [50,51]. The amount and shape of apatite formed on the titanium surface after both plasma treatments were large. Furthermore, compared to that of titanium after plasma treatment, the apatite shape was observed to be the largest in the TNS-ArP group. There have been no reports on plasma treatment affecting the ability of titanium surfaces to form apatite. However, the ability to form apatite was determined to be high on material surfaces with high surface energy, which is in agreement with the results of this study [52,53]. Furthermore, Hayakawa et al. confirmed that the apatite-forming ability of Hank’s solution correlated with bone-forming ability in animal experiments, which is also in agreement with our results [54,55,56,57].

The implant surface must be coated with protein before performing osseointegration. Albumin is known to be the most abundant plasma protein [58,59,60]. In this study, more albumin was adsorbed by performing plasma irradiation than in the TNS group, with the TNS-ArP group exhibiting the highest value. Furthermore, the nano-modified titanium surface treated with argon plasma improved the initial adhesion ability of osteoblasts and elongated the cell processes. This could contribute to the increased hydrophilicity of the nano-modified titanium surface subjected to the argon plasma treatment. The RBMCs on the nano-modified titanium surfaces treated with argon plasma exhibited higher ALP activity, calcium deposition, and osteogenesis-related gene expression than the RBMCs on the surfaces of other groups. The in vitro evaluation revealed that the nanostructure-precipitated titanium surface in the argon plasma-treated group exhibited the highest initial adhesion ability to RBMCs. Therefore, these results demonstrated that argon plasma treatment remarkably enhances the ability of nanostructures to induce hard-tissue differentiation. The in vivo analysis results were in agreement with the results of the in vitro evaluation. In this study, micro-CT and histological diagnosis exhibited increased BV/TV and Tb.Th values around the argon plasma-treated nano-modified implants. Based on these results, it can be considered that the surface of the nanostructure-precipitated implant material with argon plasma surface control substantially increased protein adsorption owing to the superhydrophilicity of the material surface and increased the bone contact rate after contacting the surface bone of the rat femur. Further studies are needed to clarify this assumption.

## 4. Materials and Methods

### 4.1. Sample Preparation

Titanium screw implants (1.2 mm external diameter and 12 mm length; Daido Steel, Osaka, Japan) and grade 2 titanium discs were purchased (15 mm diameter and 1 mm thickness; Daido Steel, Osaka, Japan). Incremental SiC abrasive sheets (800 #, 1000 #, and 1500 #) were used to polish the disks. All the samples were ultrasonically rinsed using acetone, ethanol, and deionized water (10 min each) and dried at room temperature overnight. To achieve porous, homogeneous, and uniform nanonetwork structures (TNS) on the titanium surfaces, all samples were immersed in a 10 M NaOH solution at 30 °C for 24 h. They were then repeatedly washed with ion-exchanged water (200 mL) until the conductivity of the solution reached 5 μS/cm^3^. Finally, they were dried at room temperature for an entire night. The plasma treatments on the titanium surfaces were carried out with a piezobrush^®^ PZ2 (Relyon Plazma GmbH, Regensburg, Germany). The plasma treatment was carried out using an active gas under atmospheric pressure. The low-temperature plasma treatment was carried out under irradiation at 0.2 MPa for 30 s at 10 mm (TNS-P), and the argon-based atmospheric-pressure plasma (2SLM, gas flow) treatment was carried out under irradiation at 0.1 MPa for 30 s at 10 mm (TNS-ArP). Alkali-modified titanium (TNS) discs were used as the control group, whereas TNS-P and TNS-ArP were used as the experimental groups.

### 4.2. Surface Characterization

The materials’ surface topographies were qualitatively evaluated using SEM (S-4000; Shimadzu, Kyoto, Japan) and SPM (SPM-9600; Shimadzu, Kyoto, Japan). Using a monochromatic Al Kα X-ray source, the coating composition was examined using XPS (Kratos Analytical Axis Ultra DLD electron spectrometer; Kratos Instruments, Manchester, UK). To remove surface impurities, each sample was etched with argon ions for two minutes at a rate of five nanometers per minute. Using the Multipak software tool (Multipak v9.6.1; ULVAC-PHI, Kanagawa, Japan), the elements were analyzed using the Shirley background and relative sensitivity coefficients supplied by the instrument manufacturer. To measure the contact angles and surface energies of samples from each group, one-microliter droplets of diiodomethane and deionized water were applied to their surfaces at room temperature (23–25 °C) using a surface measuring device (Drop-Master DMs-401, Kyowa Interface Science Co., Ltd., Tokyo, Japan). An interface measurement and analysis system (FAMAS, Kyowa Interface Science Co., Ltd., Tokyo, Japan) was used to analyze the images of the sample surfaces and assess each sample’s surface energy using the Owens–Wendt–Rabel–Kaelble method.

### 4.3. Protein Adsorption

The model proteins that were utilized were Bovine serum albumin (BSA) fraction V (Pierce Biotechnology, Rockford, IL, USA). Each specimen received 300 microliters of a protein solution (1 mg/mL protein in saline) pipetted onto it. After incubation for 1, 3, 6, and 24 h at 37 °C, nonadherent proteins were removed and mixed with bicinchoninic acid (Pierce Biotechnology, Rockford, IL, USA) at 37 °C for 1 h. Using a microplate reader set at 562 nm, the amount of BSA withdrawn and the total amount of BSA injected were measured. The amount of BSA that was adsorbed onto the specimen in relation to the total amount was used to compute the BSA adsorption.

### 4.4. Cell Culture

Eight-week-old SD rats’ femurs were used to harvest RBMCs (SHIMIZU Laboratory Supplies Co., Kyoto, Japan). We detailed the RBMC extraction process in our previous report [30,31,32,33,34]. The Medical Ethics Committee of Osaka Dental University in Japan granted consent for the animal tests, which were carried out in accordance with the National Animal Care Guidelines’ ethical guidelines (approval no. 21-05002). After removal from their individual flasks, the RBMCs were seeded into 24-well tissue culture plates (BD Biosciences, Franklin Lakes, NJ, USA) with titanium disks belonging to each of the three groups at a density of 4 × 10^4^ cells/well.

### 4.5. Cell Adhesion

RBMCs were seeded at an initial density of 4 × 10^4^ cells/cm^2^ onto the titanium surfaces of the three groups and allowed to attach to the surface for 1, 3, 6, and 24 h. A total of 50 μL CellTiter-Blue^®^ Reagent (Promega Corporation, Madison, WI, USA) diluted in 250 μL phosphate-buffered saline (PBS) was used to count the number of RBMC adhesions. The analysis was carried out in accordance with the manufacturer’s instructions. The cells were labeled and observed after 24 h of culture, as described in our prior research [30,31,32,33,34].

### 4.6. Quantitative Reverse Transcription Polymerase Chain Reaction (Qrt-Pcr), Alp Activity, Dna Content, and Mineralization Determination

A real-time TaqMan RT-PCR assay (Life Technologies, Carlsbad, CA, USA) was utilized to measure the expression of genes linked to osteogenesis. Using an RNeasy Mini Kit (Qiagen, Venlo, the Netherlands), the total RNA was extracted, and Prime Script RT Reagent kit (TaKaRa Bio, Shiga, Japan) was used to reverse transcribe each 10-μL aliquot of RNA sample into cDNA. To identify markers of osteogenesis, runt-related transcription factor (Runx2) was examined on day 3, the mRNA expression of ALP was examined on day 7, osteopontin (OPN) on day 14, and bone morphogenetic protein (BMP) on day 21. After 7 or 14 days of incubation, the samples were rinsed with PBS and the cells that adhered to the surface were dissolved in 300 μL of 0.2% Triton X-100 in order to measure the ALP activity. Following the manufacturer’s instructions, ALP activity was measured using an ALP luminometric enzyme-linked immunosorbent assay kit (Sigma-Aldrich, St Louis, MO, USA). The DNA content was assessed using a PicoGreen dsDNA analysis kit (Thermo Fisher Scientific, Waltham, MA, USA). In each sample, the amount of ALP was normalized to the amount of DNA. Calcium deposition in the extracellular matrix was evaluated on day 21 or 28 of culture, after dissolving in 10% formic acid. Following the manufacturer’s instructions, the calcium content was measured and computed using a Calcium E-test Kit (Wako Pure Chemical Industries Ltd., Osaka, Japan).

### 4.7. Intracellular ROS Levels in Rbmcs

The CellROX^®^ oxidative stress reagent (C10422, Thermo Fisher Life Technologies Ltd., Tokyo, Japan) was used to measure intracellular ROS levels. The cells were rinsed three times with PBS before being incubated for 30 min at 37 °C in a medium containing 5 μM CellROX^®^ oxidative stress reagents. Trypsinization was used to collect RBMCs, which were then transferred to a 24-well plate. ROS levels in the RBMCs on titanium discs from the three groups were stained and observed using confocal laser scanning microscopy (LSM700; Zeiss, Oberkochen, Germany).

### 4.8. Simulated Body Fluid (SBF) Test

TNS, TNS-P, and TNS-ArP specimens were soaked in 50 mL of SBF solution at 35–38 °C for 2 weeks to assess the production of apatite on the titanium surface (negative control, Ti). Ion concentrations in the SBF solution were similar to those found in plasma from human blood [48,49]. Following their immersion in the SBF solution, each specimen underwent multiple rinses in distilled water. SEM, FTIR spectroscopy (FTIR; Spectrum One, Perkin Elmer, Massachusetts, USA), and thin-film X-ray powder diffractometry (TF-XRD; RINT-2500, Rigaku Co., Tokyo, Japan) were used to examine morphological changes on the specimens’ surfaces.

### 4.9. Animal Model and Surgical Procedures

In this study, eight-week-old male SD rats (Shimizu Laboratory Supplies Co., Kyoto, Japan) were utilized. The rats were randomly divided into three groups of eight rats each. The surgical techniques employed in this investigation were previously reported [30,31,32,33,34]. Following general anesthesia and surgical cleaning, a 10-mm longitudinal incision was made along the medial side of the right hind leg’ s knee joint. Following that, the patella and extensors were dislocated to expose the distal femurs. Using a dental burr and sterile saline irrigation, a 1.2-mm hole was bored into the intercondylar notch. The titanium screw implants were inserted into the channels that had been made, the knee joint was repaired, and the incision was sutured. To prevent postoperative infection and postoperative pain, gentamicin (1 mg/kg) and buprenorphine (0.05 mg/kg) were injected for 3 days postoperatively.

### 4.10. In Vivo Argon Plasma-Induced Bone Differentiation on the TNS-Modified Titanium Surface

The right femurs, including the implants, were immediately placed in a cold saline solution and scanned with a micro-computed tomography (micro-CT) scanner (SkyScan1275, Bruker, Billerica, MA, USA) operating at 70 kV and 118 mA; the isotropic voxel size was 10 μM in all spatial directions. The implant and surrounding tissue were rebuilt and examined using morphometric software (TRI/3D-BON; Ratoc System Engineering, Tokyo, Japan) after tomographic capture. The ROI was defined as the 500-m wide area of bone around the implants from 2 mm below the greatest point of the growth plate to the distal 100 slices. Within the ROI, the BV/TV and mean Tb.Th were calculated. The femoral specimens were utilized to make undecalcified histological sections after micro-CT scanning. The specimens were fixed in 70% ethanol for 7 days before being immersed in Villanueva bone stain solution. Using a digital microscope (BZ-9000; Keyence Co., Osaka, Japan), sections were histomorphometrically analyzed. A confocal laser scanning microscope (LSM700; Zeiss, Oberkochen, Germany) was used for fluorescence microscopy.

### 4.11. Statistical Analyses

The in vitro evaluation was repeated four times. The femurs of eight rats from each group were used for the in vivo experiments. All quantitative data are presented as means ± SD. The results were analyzed by one-way analysis of variance and Tukey’s post hoc test using GraphPad Prism 8.0 software (GraphPad Prism, San Diego, CA, USA). The level of statistical significance was established at *p* < 0.05, with lower *p* values considered highly significant.

## 5. Conclusions

The experimental results with argon plasma-treated titanium surfaces with deposited nanostructures show that carbon contaminants were removed while maintaining the reticulate nanostructures deposited by concentrated alkali treatment, and the surface energy was increased by increasing the reactive oxygen and nitrogen species on the surface of the material. Reactive oxygen species on titanium treated with argon plasma reduced the oxidative stress on RBMCs, creating a favorable environment for cell growth. The highest apatite formation was observed in the argon plasma-treated group in the experiment using SBF. The results of this work demonstrated the ability of the nanostructured titanium surface to induce hard tissue differentiation enhanced by the argon plasma treatment, which could be useful for new bone formation around implant sites.

## Data Availability

No new data were created or analyzed in this study. Data sharing is not applicable to this article.

## References

[1] Moradian-Oldak J., Wen H.B., Schneider G.B., Stanford C.M. (2006). Tissue engineering strategies for the future generation of dental implants. Periodontology 2000.

[2] Branemark P.I. (1983). Osseointegration and its experimental background. J. Prosthet. Dent..

[3] Carlos O., Alejandro D. (2012). Titanium as a Biomaterial for Implants. J. Arthroplast..

[4] John W.N. (2020). Titanium Alloys for Dental Implants: A Review. Prosthesis.

[5] Romeo E., Storelli S. (2012). Systematic review of the survival rate and the biological, technical, and aesthetic complications of fixed dental prostheses with cantilevers on implants reported in longitudinal studies with a mean of 5 years follow-up. Clin. Oral Implant. Res..

[6] Ogawa T., Nishimura I. (2003). Different bone integration profiles of turned and acid-etched implants associated with modulated expression of extracellular matrix genes. Int. J. Oral Maxillofac. Implant..

[7] Ogawa T., Ozawa S., Shih J.H., Ryu K.H., Sukotjo C., Yang J.M., Nishimura I. (2000). Biomechanical evaluation of osseous implants having different surface topographies in rats. J. Dent. Res..

[8] Schwartz Z., Lohmann C.H., Oefinger J., Bonewald L.F., Dean D.D., Boyan B.D. (1999). Implant surface characteristics modulate differentiation behavior of cells in the osteoblastic lineage. Adv. Dent. Res..

[9] Takeuchi K., Saruwatari L., Nakamura H.K., Yang J.M., Ogawa T. (2005). Enhanced intrinsic biomechanical properties of osteoblastic mineralized tissue on roughened titanium surface. J. Biomed. Mater. Res. A.

[10] Xuanyong L., Paul K.C., Chuanxian D. (2004). Surface modification of titanium, titanium alloys, and related materials for bio-medical applications. Mater. Sci. Eng. R. Rep..

[11] Ulrike D. (2003). The surface science of titanium dioxide. Surf. Sci. Rep..

[12] Vandrovcová M., Bačáková L. (2011). Adhesion, growth and differentiation of osteoblasts on surface-modified materials developed for bone implants. Physiol. Res..

[13] Komasa S., Taguchi Y., Nishida H., Tanaka M., Kawazoe T. (2012). Bioactivity of nanostructure on titanium surface modified by chemical processing at room temperature. J. Prosthodont. Res..

[14] Fujino T., Taguchi Y., Komasa S., Sekino T., Tanaka M. (2014). Cell differentiation on nanoscale features of a titanium surface: Effects of deposition time in NaOH solution. J. Hard Tissue Biol..

[15] Zhang H., Komasa S., Mashimo C., Sekino T., Okazaki J. (2017). Effect of ultraviolet treatment on bacterial attachment and osteogenic activity to alkali-treated titanium with nanonetwork structures. Int. J. Nanomed..

[16] Kasuga T., Hiramatsu M., Hoson A., Sekino T., Niihara K.K. (1999). Titania nanotubes prepared by chemical processing. Adv. Mater..

[17] Oliveira M., Radi P., Reis D., Reis A. (2021). Titanium Bioactive Surface Formation Via Alkali and Heat Treatments for Rapid Osseointegration. J. Mater. Res..

[18] Mohamed M., Samy E., Eman E. (2022). Effect of alkaline treatment with sodium hydroxide on wettability and bioactivity of some commercial dental implants: An in-vitro study. Tanta. Dent. J..

[19] Umehara H., Doi K., Oki Y., Kobatake R., Makihara Y., Kubo T., Tsuga K. (2020). Development of a novel bioactive titanium membrane with alkali treatment for bone regeneration. Dent. Mater. J..

[20] Seyda V., Niko K., Claus E. (2012). Investigation of aging processes of Ti-6Al-4 V powder material in laser melting. Phys. Procedia.

[21] Wang K. (1996). The use of titanium for medical applications in the USA. Mater. Sci. Eng. A.

[22] Zhao G., Schwartz Z., Wieland M., Rupp F., Geis-Gerstorfer J., Cochran D.L., Boyan B.D. (2005). High surface energy enhances cell response to titanium substrate microstructure. J. Biomed. Mater. Res. A.

[23] Wang S., Zhang Y., Abidi N., Cabrales L. (2009). Wettability and surface free energy of graphene films. Langmuir.

[24] Aita H., Hori N., Takeuchi M., Suzuki T., Yamada M., Anpo M., Ogawa T. (2009). The effect of ultraviolet functionalization of titanium on integration with bone. Biomaterials.

[25] Ohko Y., Hashimoto K., Fujishima A. (1997). Kinetics of photocatalytic reactions under extremely low-intensity UV illumination on titanium dioxide thin films. J. Phys. Chem. A.

[26] Park J.H., Schwartz Z., Olivares-Navarrete R., Boyan B.D., Tannenbaum R. (2011). Enhancement of surface wettability via the modification of microtextured titanium implant surfaces with polyelectrolytes. Langmuir.

[27] Elias C.N., Oshida Y., Lima J.H., Muller C.A. (2008). Relationship between surface properties (roughness, wettability and morphology) of titanium and dental implant removal torque. J. Mech. Behav. Biomed. Mater..

[28] Rupp F., Scheideler L., Rehbein D., Axmann D., Geis-Gerstorfer J. (2004). Roughness induced dynamic changes of wettability of acid etched titanium implant modifications. Biomaterials.

[29] Iwasa F., Hori N., Ueno T., Minamikawa H., Yamada M., Ogawa T. (2010). Enhancement of osteoblast adhesion to UV-photofunctionalized titanium via an electrostatic mechanism. Biomaterials.

[30] Hatoko M., Komasa S., Zhang H., Sekino T., Okazaki J. (2019). UV treatment improves the biocompatibility and antibacterial properties of crystallized nanostructured titanium surface. Int. J. Mol. Sci..

[31] Ujino D., Nishizaki H., Higuchi S., Komasa S., Okazaki J. (2019). Effect of plasma treatment of titanium surface on biocompatibility. Appl. Sci..

[32] Matsumoto T., Tashiro Y., Komasa S., Miyake A., Komasa Y., Okazaki J. (2021). Effects of surface modification on adsorption behavior of cell and protein on titanium surface by using quartz crystal microbalance system. Materials.

[33] Takao S., Komasa S., Agariguchi A., Kusumoto T., Pezzotti G., Okazaki J. (2020). Effects of plasma treatment on the bioactivity of alkali-treated ceria-stabilised Zirconia/Alumina nanocomposite (NANOZR). Int. J. Mol. Sci..

[34] Komasa S., Takao S., Yang Y., Zeng Y., Li M., Yan S., Zhang H., Komasa C., Kobayashi Y., Nishizaki H. (2020). Effects of UV treatment on ceria-stabilized zirconia/alumina nanocomposite (NANOZR). Materials.

[35] Silva I., Barreto A., Seixas R., Paes P., Lunz J., Thire R., Jardim P. (2023). Novel Strategy for Surface Modification of Titanium Implants towards the Improvement of Osseointegration Property and Antibiotic Local Delivery. Materials.

[36] Canullo L., Genova T., Tallarico M., Gautier G., Mussano F., Botticelli D. (2016). Plasma of argon affects the earliest biological response of different implant surfaces: An in vitro comparative study. J. Dent. Res..

[37] Wang L., Wang W., Zhao H., Liu Y., Liu J., Bai N. (2020). Bioactive Effects of Low-Temperature Argon–Oxygen Plasma on a Titanium Implant Surface. ACS Omega.

[38] Canullo L., Genova T., Wang H.L., Carossa S., Mussano F. (2017). Plasma of argon increases cell attachment and bacterial decontamination on different implant surfaces. Int. J. Oral Maxillofac. Implant..

[39] Coelho P.G., Giro G., Teixeira H.S., Marin C., Witek L., Thompson V.P., Tovar N., Silva N.R. (2012). Argon-based atmospheric pressure plasma enhances early bone response to rough titanium surfaces. J. Biomed. Mater. Res. A.

[40] Carossa M., Cavagnetto D., Mancini F., Balma A., Mussano F. (2022). Plasma of Argon Treatment of the Implant Surface, Systematic Review of In Vitro Studies. Biomolecules.

[41] Canullo L., Rakic M., Corvino E., Burton M., Krumbeck J., Prem A., Ravida A., Ignjatovic N., Sculean A., Menini M. (2023). Effect of argon plasma pre-treatment of healing abutments on peri-implant microbiome and soft tissue integration: A proof-of-concept randomized study. BMC Oral Health.

[42] Canullo L., Donato A., Savadori P., Radovanovic S., Lacono R., Rakic M. (2023). Effect of argon plasma abutment activation on soft tissue healing: RCT with histological assessment. Clin. Implant. Dent. Relat. Res..

[43] Jee H.J., Kim H.J., Kim A.J., Bae Y.S., Bae S.S., Yun J. (2009). UV light induces premature senescence in Akt1-null mouse em-bryonic fibroblasts by increasing intracellular levels of ROS. Biochem. Biophys. Res. Commun..

[44] Scharffetter-Kochanek K., Wlaschek M., Brenneisen P., Schauen M., Blaudschun R., Wenk J. (1997). UV-induced reactive ox-ygen species in photocarcinogenesis and photoaging. Biol. Chem..

[45] Tang K., Zhan J.C., Yang H.R., Huang W.D. (2010). Changes of resveratrol and antioxidant enzymes during UV-induced plant defense response in peanut seedlings. J. Plant Physiol..

[46] Kokubo T., Takadama H. (2006). How useful is SBF in predicting in vivo bone bioactivity?. Biomaterials.

[47] Amir Z. (2014). Relationship between in vitro apatite-forming ability measured using simulated body fluid and in vivo bioactivity of biomaterials. Mater. Sci. Eng. C.

[48] Bharati S., Mithiles K.S., Basu D. (2005). Hydroxyapatite coating by biomimetic method on titanium alloy using concentrated SBF. Bulletin. Mater. Sci..

[49] Gu Y.W., Khor K.A., Cheang P. (2003). In vitro studies of plasma-sprayed hydroxyapatite/Ti-6Al-4V composite coatings in simulated body fluid (SBF). Biomaterials.

[50] Liang F., Zhou L., Wang K. (2003). Apatite formation on porous titanium by alkali and heat-treatment. Surf. Coat. Tech..

[51] Jonášová L., Muller F.A., Helebrant A., Strnad J., Greil P. (2002). Hydroxyapatite formation on alkali-treated titanium with different content of Na+ in the surface layer. Biomaterials.

[52] Chen X.B., Li Y.C., Hodgson P.D., Wen C. (2009). The importance of particle size in porous titanium and nonporous counter-parts for surface energy and its impact on apatite formation. Acta Biomater..

[53] Hieda J., Sakaguchi A., Nakano M., Akasaka H., Ohtake N. (2019). Relationships between surface energy and charge of sur-face-modified titanium and HAp formation. Appl. Surf. Sci..

[54] Hayakawa S., Matsumoto Y., Uetsuki K., Shirosaki Y., Osaka A. (2015). In vitro apatite formation on nano-crystalline titania layer aligned parallel to Ti6Al4V alloy substrates with sub-millimeter gap. J. Mater. Sci. Mater. Med..

[55] Hayakawa S., Okamoto K., Yoshioka T. (2019). Accelerated induction of in vitro apatite formation by parallel alignment of hydrothermally oxidized titanium substrates separated by sub-millimeter gaps. J. Asian Ceram. Soc..

[56] Tsutsumi Y., Nishimura D., Doi H., Nomura N., Hanawa T. (2009). Difference in surface reactions between titanium and zirconium in Hanks’ solution to elucidate mechanism of calcium phosphate formation on titanium using XPS and cathodic polarization. Mater. Sci. Eng. C.

[57] Wei Y., Jing Q., Ling X., Fu Z. (2009). Corrosion behaviors of TiO2 nanotube layers on titanium in Hank’s solution. Biomed. Mater..

[58] Raphel J., Karlsson J., Galli S., Wennerberg A., Lindsay C., Haugh M.G., Pajarinen J., Goodman S.B., Jimbo R., Andersson M. (2016). Engineered protein coatings to improve the osseointegration of dental and orthopaedic implants. Biomaterials.

[59] Terheyden H., Lang N.P., Bierbaum S., Stadlinger B. (2012). Osseointegration–communication of cells. Clin. Oral Implant. Res..

[60] Zhong J., Li X., Yao Y., Zhou J., Cao S., Zhang X., Jian Y., Zhao K. (2022). Effect of acid-alkali treatment on serum protein adsorption and bacterial adhesion to porous titanium. J. Mater. Sci. Mater. Med..

